# Organisational capabilities, outcomes, and benefits of trigger finger release surgery in primary care: a service evaluation study

**DOI:** 10.3399/BJGPO.2023.0031

**Published:** 2023-11-01

**Authors:** Fahad Rizvi, Chen Wei Rong Ryan, Kong Amos Ethan, Wong Chun Pui Joshua, Neal Khambhayata, Dhriti Arya, Tariq Kapasi, Philippe B Wilson

**Affiliations:** 1 NHS Willows Health, Leicester, UK; 2 NHS Leicester, Leicestershire and Rutland Integrated Care Board, Leicester, UK; 3 University of Leicester, Leicester, UK; 4 Medical Technologies Innovation Facility, Clifton Campus, Nottingham Trent University, Nottingham, UK

**Keywords:** general surgery, orthopaedics, rheumatology, primary health care

## Abstract

**Background:**

Trigger finger is a common hand condition in which a finger is unable to fully extend owing to a thickening of the tendon and its sheath, causing the finger to lock in a bent position.

**Aim:**

To assess the viability of carrying out trigger finger surgeries in NHS primary care in terms of clinician and patient acceptance, experience, and outcomes; and operational requirements of this service for wider application.

**Design & setting:**

A pilot study for a new service in primary care in Leicestershire, UK.

**Method:**

A total of 214 trigger finger release surgery procedures were carried out between 22 August 2019 and 25 October 2022 by a single hand surgeon in a single primary care surgery. Data were analysed using information from SystmOne, which is a patient database linked with the NHS.

**Results:**

Thirty-two cases out of 214 (15.0%) experienced a wait time of <10 days from the GP referral to the first outpatient appointment (OPA). Out of 214 procedures, there were 26 (12.1%) postoperative complications that required follow-up action. Of the total number of cases where postoperative complications were identified, 13 cases required further follow-up action, while the remaining 13 did not have any identifiable further follow-up action and were regarded as closed cases thereafter.

**Conclusion:**

Trigger finger release surgery in primary care offers an opportunity to reduce pressures on secondary care orthopaedic referrals, as well as offering patients faster and effective surgical treatment while utilising fewer NHS resources.

## How this fits in

There are huge challenges in addressing long orthopaedic waiting lists in UK secondary care. Qualified surgeons in the primary care sector can help to reduce waiting times by completing standard and non-complex cases in this setting. This article describes a clinic set-up, including operational requirements and positive patient outcomes for trigger finger surgeries, carried out within a primary care network in Leicester, UK.

## Introduction

Trigger finger is defined as tenosynovitis in the flexor sheaths of the fingers and thumb.^1^ It can arise from repetitive gripping actions that can cause narrowing of the flexor pulley sheaths in the hands.^2^ Progressively, the hypertrophy and inflammation of the retinacular sheath can restrict motion of the flexor tendons of the affected hand.^3^

Trigger finger can impact on quality of life.^4^ It is characterised by symptoms of the condition, such as stiffness, locking, and pain,^5^ which are frequently reported at the metacarpophalangeal joints.^6^

Management can include conservative measures such as activity modification and analgesia for pain relief in the form of non-steroidal anti-inflammatory drugs (NSAIDs).^7^ However, steroid injections carried out under local anaesthetic are often recommended as the first-line treatment,^5^ with referral for secondary care opinion if the patient expresses a preference for surgical release or if symptoms fail to resolve after steroid injections.^8^ Schubert and colleagues have described demographics of trigger finger occurrence and corticosteroid injection therapy in a retrospective review. They identified a considerably higher proportion of female cases (71% versus 29% for males), with women reporting symptoms at an average age of 58 years compared with 62 years for men. Prevalence of diabetes in the cohort was 22% and no correlation was observed between trigger digit and dominance of hand, while the right index and thumbs were the most commonly affected digits.^9^

Surgery in the form of either an open or percutaneous release of the affected finger is the current recommended method to treat trigger finger.^10^ This highly successful procedure is well recognised as the most effective treatment for trigger finger.^11^

However, current waiting times for OPAs in Leicestershire alone rest at 17 weeks for OPA and 22 weeks for treatment.^12^ This article describes a primary care service developed with an average patient waiting time of 4 weeks in comparison, as well as the operational and postoperative outcomes associated with the running of this service.

### Aims

This study aimed to examine the clinical outcomes of trigger finger release surgeries conducted in a primary care setting in order to understand the impact of carrying out such procedures in the community. Furthermore, the operational requirements and learnings from developing the service are detailed in order to provide other organisations with the opportunities to expand this offering into their own service distributions.

This study was particularly interested in establishing current waiting times from a patient’s first referral from their GP to the first OPA at the trigger finger clinic, and the subsequent lead time from the first OPA at the trigger finger clinic to the trigger finger release surgery itself.

The postoperative outcomes of these procedures were also scrutinised by analysing a range of related parameters such as the percentage of postoperative complications, the nature of such complications (for example, pain, infection, swelling), and the follow-up actions required for each case.

## Method

A total of 214 trigger finger release surgery procedures were carried out between 22 August 2019 and 25 October 2022. The procedures, undertaken by a single hand surgeon in a single primary care surgery in Leicester, UK, were scrutinised using data from SystmOne, which is a patient database linked with the NHS. The procedures were carried out on 107 male and 107 female patients. For each procedure, the following were recorded: the hand operated on (left or right); and the finger of that hand that was operated on (thumb, index, middle, ring, little). The date of the patient’s first GP referral was identified, together with the date of the first OPA clinic with the hand surgeon. Before this, the patient had the opportunity to have a telephone discussion with their GP and the surgeon to discuss referral if they wished. Alternatively, the patient had the opportunity to be directly referred for their first OPA, which thereby delivers the fast turnaround times for initial consultation and completion of the procedure. It was also noted if there were any corticosteroid injections administered for each case and, if so, the date on which it was administered. Finally, the date of each trigger finger release surgery procedure was recorded.

Following this, any postoperative follow-ups were identified on SystmOne. If no further data were identified after the date of the procedure, it was assumed that there would be no patient or clinician initiated follow-up and the case was closed. However, if further data were found after the date of the procedure, then this would be marked as a follow-up and checked for any postoperative complication. The total number of postoperative complications were recorded, and for each complication the following were noted: the nature of the complication (for example, pain, infection, swelling); the date that it was identified; and whether any follow-up action was taken (for example, referral to secondary care, steroid injection, antibiotics, and so on), and, if so, the date of action taken.

## Results

The average patient spent 28 days waiting for an OPA clinic appointment with the hand surgeon (Table 1). This data is spread out with moderate consistency given the standard deviation of 27.8 days. It should be noted that 32 cases out of 214 (15.0% of all cases) experienced a wait time of <10 days from the GP referral to the first OPA, with two cases being seen on the same day by the hand surgeon as the GP referral itself. There were only six cases (<3% of all cases) that experienced a wait time of >100 days and this was attributed to patient choice of having the procedure at a later date owing to their commitments. Essentially, this highlights the capacity for a quick referral from the GP to the first OPA with the hand surgeon, which could save precious time for patients.

**Table 1.** A summary of mean and median waiting times during the study

Mean time taken from GP referral to first OPA, days	28.0
Median time taken from GP referral to first OPA, days	23.0
Standard deviation (in days) of time taken from GP referral to first OPA	27.8
Mean time taken from first OPA to surgery, days	51.3
Median time taken from first OPA to surgery, days	14.0
Standard deviation (in days) of time taken from first OPA to surgery	90.7

OPA = outpatient appointment

The data for the time taken from the first OPA clinic to the actual surgery itself was more dispersed, with a standard deviation of 90.7 days. Given the mean time from the first OPA to the surgery (51.3 days), the average person would likely wait <2 months after the first OPA clinic before undergoing surgery.

Given that standard practice guidance, as described earlier, indicates two injections before operation, patients had already experienced injections previously and indicated a preference for surgery. Therefore, based on a combination of patient choice as well as clinical guidance, 39 cases out of 214 had opted for an initial treatment of steroid injections at the first OPA before eventually opting for surgery. This could possibly have lengthened the time recorded from the first OPA to the surgery, thus affecting the obtained data. Furthermore, personal availability for the surgery is a contributing factor, as with the possibility that some patients could have preferred to see how symptoms developed before the surgery.

It should be highlighted that 71 cases (33.2% of all cases) experienced less than a 10-day waiting time between the first OPA and the surgery itself, with 18 cases (8.41% of all cases) undergoing surgery on the same day as the first OPA itself. This was due to patients’ previous experience of trigger release, failed steroid treatment, and opportunity to get the surgery on the same day, if not they were offered a trigger-release procedure within 4 weeks. This suggests that there was a capacity for almost one-third of all cases to be seen at a reasonably short notice, which could suggest the efficiency and time-management advantages of conducting trigger finger release surgery in primary care.

**Figure 1.**
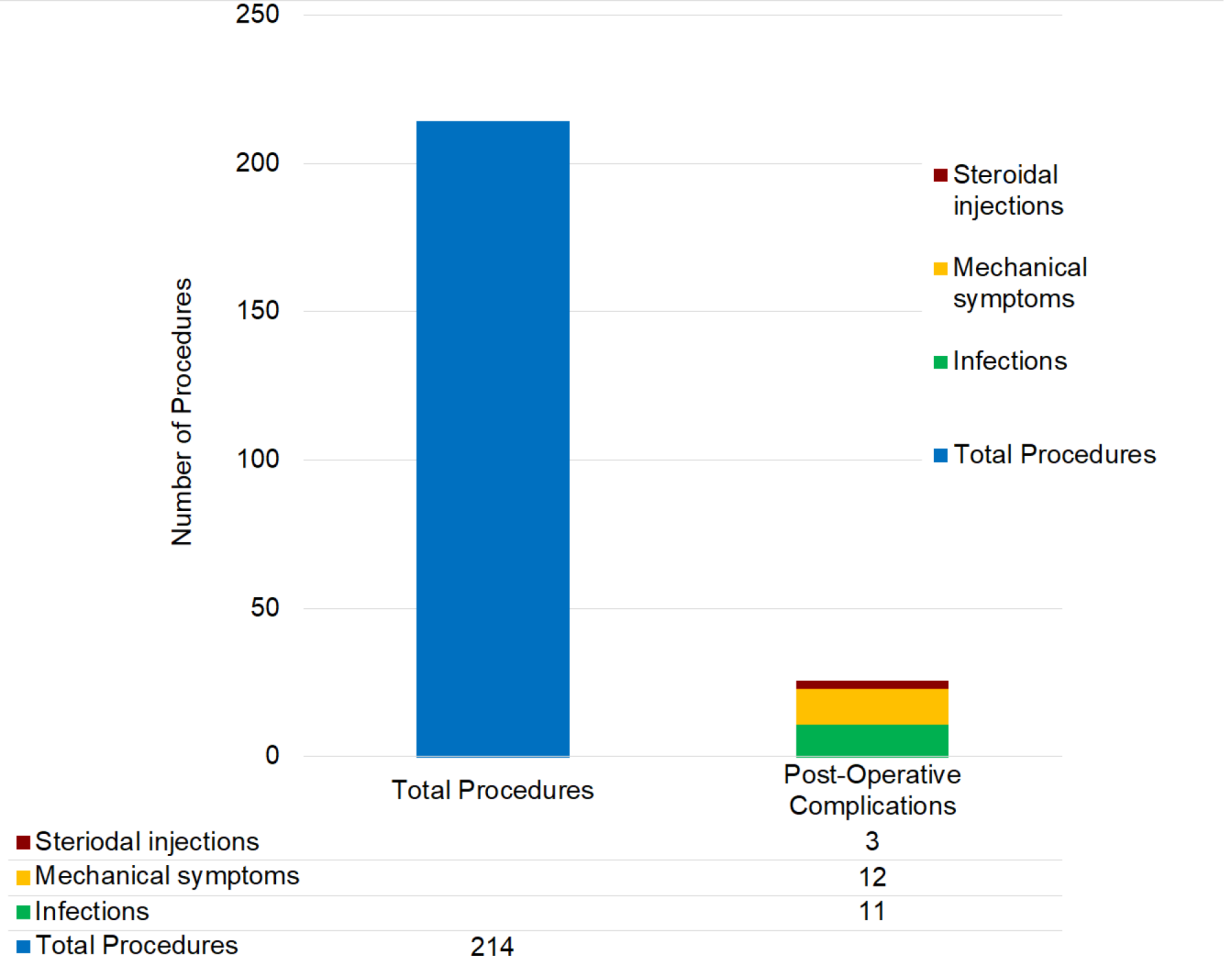
Classification of postoperative complications compared with total procedures carried out within the study. Steroid injection was for tenosynovitis following trigger release. Mechanical symptoms were stiffness, swelling, and scar inflammation. Infection referred to superficial infection, which was treated with normal dose (500 mg qds for 7 days) flucloxacillin, or deep infection, which required a surgical washout for tendon sheath infection. In this case series, only one patient required surgical intervention.

Out of the total of 214 unique procedures carried out, there were 26 postoperative complications that required follow-up action (Figure 1), which comprised 12.1% of the total number of procedures carried out. Of the total number of cases where postoperative complications were identified, 13 cases required further follow-up action, while the remaining 13 did not have any identifiable further follow-up action, and were regarded as closed cases thereafter. Eleven suspected postoperative infections were identified, where signs and symptoms such as pain, swelling, and the development of pus were reported. Out of all 214 cases performed, this equates to 5.1%. Twelve cases were identified as depicting signs and symptoms suggestive of postoperative mechanical symptoms (for example, stiffness, locking). This equates to 5.6% of the total number of procedures performed, as shown in Figure 1.

Three cases required steroid injections after the procedure, which made up 1.4% of all 214 cases. One case was identified as requiring further surgical intervention after the trigger finger release surgery. This represented 0.5% of all the 214 procedures performed.

These data suggested that there are reasonably low chances of postoperative complications developing, with the vast majority of cases not requiring further postoperative action. Given that complications for trigger finger release surgery can be as high as 31%,^13^ the notably lower postoperative complication rate of 12.1% in the present study has indicated a potential benefit of conducting trigger finger release surgery in primary care. Furthermore, a 5.7% risk of peripheral nerve damage with percutaneous release has been reported,^13^ hence many surgeons favouring open release as an alternative.

The number of operations carried on each hand (left or right) and the number of different fingers involved (that is, thumb, index, middle, ring, or little) are described in Table 2. Notably, 55.1% of procedures were carried out on the right hand, with the middle and ring fingers being the most likely fingers to be operated on. Conversely, the thumb was operated on in only 5.1% of cases.

**Table 2.** The number of operations carried out on the hand and the individual fingers (*n* = 214)

	Left hand	Right hand
Total on each hand	96	118
Thumb	3	8
Index	11	9
Middle	39	45
Ring	36	44
Little	7	12

### Service operationalisation and delivery

#### Rationale

The service was designed to leverage the existing expertise in primary care and simultaneously aimed to reduce waiting lists at the local acute trust. Indeed, a 2015 review by Hussenbux and coworkers considered the potential as well as output impact of interdisciplinary triage-and-treat services at the primary care interface for musculoskeletal conditions.^14^ Across the 23 studies identified, ≥72% of patients could be managed at an earlier stage through intermediate care pathways, leading directly to a 20%–60% reduction in orthopaedic referral rates. Furthermore, there have been further references to such interfacial services for the reduction in orthopaedic outpatient waiting lists, specifically in consideration of the shifting policy landscape within the UK NHS.^15^ Ferguson and Cooke^15^ assessed the NHS Bath and North East Somerset Orthopaedic Interface Service, where an intermediate service resulted in only 21% of patients being referred to secondary care, the remainder being successfully managed within primary care settings.

#### The setting and integration within the integrated care model

At the time of the study, the referral areas were Leicester City clinical commissioning group (CCG) and East Leicestershire and Rutland CCG, covering more than 1.2 million patients. The caseload described within this study accounted for 50% of the cases, where the remaining 50% of referrals were undertaken by secondary care. All referrals were made by the referral service scheme, which is a standard NHS practice where this offering was integrated into the opportunity. Furthermore, patients were referred to the service as part of the standard clinical pathway including previous iterations of corticosteroid injections before surgical referral.

#### The approach

The methodology was based on the concept of GP retraining where a number of clinicians in primary care had sought additional training in specialty fields that they are able to leverage. In this instance, a GP with surgical specialism and qualifications collaborated with a local consultant hand surgeon in the acute care trust in order to establish this service. The local GP surgical specialist and consultants working in the primary care setting were allocated 250 procedures per year in order to achieve the availability described.

Surgeries were carried out in existing, purpose-built minor surgery units in primary care, with ≥15 air changes per hour. The facility benefitted from a Service Level Agreement with the local CCG at the time. Operationally, the service was able to run at just above half the current NHS tariff for such procedures, as the economies of scale within the primary care units allowed for savings on porters, multiple theatre assistants, large administrative teams, and estates and overhead costs. Indeed, the current NHS tariff to secondary care for such procedures is marginally over £1000 per case, whereas the service described within this study was able to deliver such procedures at a 40% cost-saving, thereby £600. In comparison, the team used to deliver this service consisted of the following: GP specialist or consultant, theatre assistant (mid-band nurse), district nurse (for follow-ups), and an administrative colleague to arrange appointments and additional clerical tasks.

Patients were called at the surgery slot and in most cases would be discharged from the surgery within 30 minutes of arrival, thereby avoiding unnecessary waits, overcrowding, and patient anxiety. Follow-up was carried out by the district nurse team for stitch removal and benfitted from open-access patient initiated follow-up appointments (PIFUs) for any concerns or queries relating to the service. This avoided patient anxiety concerning additional clinician contact and reduced likelihood of emergency department visits given the open-access nature of the PIFUs as an alternative.

#### Evaluation and postoperative care

Patients received a hand evaluation questionnaire 6-weeks post-surgery in order to assess their recovery and capture any concerns, with the option of scheduling a follow-up appointment. On patient-initiated follow-up, the surgeon would receive an automated email with any additional information, such as wound photographs or patient notes, and scheduled a slot to call the patient. This would be carried out as a same-day service either by audio or video call. The surgeon would discuss with the patient their preference and a face-to-face appointment would be set up if clinically required or requested by the patient.

The development of this service — with demonstrable ease of access for patients, as well as the open and accessible follow-up opportunities, in addition to the improved time and financial considerations — indicated an overall benefit for implementation. Indeed, of all the referrals triaged to primary care, locally 90% of trigger-release interventions were carried out in primary care with 10% referred to the acute trust as the presentation was not commensurate to trigger finger, instead being a related condition such as Dupuytren’s contracture, flexor or extensor injury, ganglions, and arthritis.

## Discussion

### Summary

This study has suggested potential benefits of conducting trigger finger release surgery in primary care with regard to waiting times and the quality of the procedures undertaken. These include the quality of the procedures undertaken; the ease of access of initial and follow-up appointments for patients; improved waiting times; and cost-saving to the NHS as compared with referral to secondary services.

### Strengths and limitations

While this study was carried out as a pilot for a new service opportunity, the delivery and operationalisation of the service as well as patient outcomes suggest a strong opportunity for service extension to further primary care centres. The number of participants (*n* = 214) may be considered relatively low (a result of the service being based in a single PCN); however, this is pertinent when considered in the wider context of evaluating the delivery of this new service for potential extension to other community-based offerings.

### Comparison with existing literature

Trigger finger release surgery in primary care has been found to have success rates of more than 90% in a recent study.^11^ Moreover, of the 214 procedures undertaken in the present study, only 12.1% resulted in postoperative complications; this is considerably lower than the up to 31% noted previously.^13^ Futhermore, of the 12.1% postoperative complications, only half required follow-up actions, arguably indicating that a total of 6% of all cases performed justified treatment. This suggests that risk factors and prevalence of such complications are not increased by undertaking prcoedures in a primary care setting; instead an improvement in availability of such procedures to patients is seen, with a marked improvement in postoperative complication rates. The ease of contacting the operating surgeon also gave confidence to patients. The patients were provided phone numbers to contact without the need to return to their GPs or attend busy emergency departments in working hours. They had open access to follow-ups and with a variety of same-day consultation options, such as phone, video call, email, or face to face, as per the patient’s convenience and request.

A 2019 study carried out at the University of Utah considered cost implications of surgical interventions in operating rooms *versus* those carried out in minor theatres or procedure clinics, providing a similar comparison between the secondary and primary care intervention environments in the UK. Principally, and considering the healthcare funding landscape differences in the US, the study identified a 221% cost increase from carrying out trigger finger release surgeries in operating theatres as opposed to procedural clinics.^16^ The British Society for the Surgery of the Hand (BSSH) in their *Evidence for Surgical Treatment* brief^17^ carried out a systematic review and meta-analysis of the following treatment options described in the literature: corticosteroid injections; open surgery; and percutaneous release. Of the trials analysed, the 2001 study by Gilberts *et al* followed percutaneous release and open surgery patients within 12 weeks of the procedure and did not identify any difference in recurrence rates, which were low for both procedures.^18^ Furthermore, a 2015 randomised study by Sato *et al* compared open A1 pulley release, percutaneous release and steroid injections in 150 digits over 6-month follow-ups in a population aged >15 years. While no complications were noted, equivalent outcomes in both percutaneous and open A1 pulley release surgery were reported.^19^ Atthakomol and colleagues recently reported results of a 12-year retrospective observational study with a 2.39% recurrence rate over 841 fingers. More than three previous steroid injections and a history of manual labour were identified as independent predictors of recurrence.^20^

### Implications for research and practice

A number of cases were identified and operated on the same day, with successful clinical outcomes such as low postoperative complication rates. These are suggestive of the high efficiency and standards associated with this practice in primary care, which could contribute to overall patient satisfaction and their quality of life with early return to work. In the future, this practice has the potential to reduce secondary care pressures should more trigger finger release surgeries be managed in a primary care setting. Furthermore, procedures carried out in primary care, such as open release, can also allow for initial indentification of less common conditions such as traumatic or atraumatic ruptures of the flexor digitorum profundus.^21^

Of the good practice points identified in the BSSH report, recommendations to practitioners centred around referral to secondary care for surgeries. The present study has suggested a real opportunity to relieve pressures on otheopaedic clinics in secondary care by offering such procedures in a primary care setting.

## Data

The dataset relied on in this article is available from the corresponding author on reasonable request.
